# Adolescent pregnancy persists in Nigeria: Does household heads’ age matter?

**DOI:** 10.1371/journal.pgph.0003212

**Published:** 2024-05-15

**Authors:** Christian Otado Mbulu, Li Yang, Gwenyth R. Wallen

**Affiliations:** Department of Health and Human Services, National Institutes of Health, Clinical Center, Translational Biobehavioral and Health Disparities Branch, Bethesda, Maryland, United States America; Augusta University, UNITED STATES

## Abstract

About 700,000 pregnant youths die each year in developing countries. To determine whether the persistent adolescent pregnancy in Nigeria between 2013 and 2018 was influenced by proximal factors, particularly household head age, we carried out a cross-sectional study on adolescent girls that participated in the 2018 Nigeria Demographic and Health Survey (NDHS). Age of first birth, residence type, age, and gender of household heads was collected using a 2018 standardized NDHS. Multiple logistic regression was performed to test for associations. We analyzed 8,448 adolescents who had experienced pregnancy during the study period. The results demonstrated that girls with male household heads aged 45 and older have lower odds of adolescent pregnancy (OR = 0.619; 95% CI = 0.447, 0.856; *p* = 0.004 compared to those with female household heads in the same age group. Girls with male household heads in three younger age groups have higher odds of adolescent pregnancy than those with female household heads in the corresponding age group (15–24: OR = 1.719, 95% CI = 1.042, 2.835, p = 0.034; 25–34: OR = 4.790, 95% CI = 1.986, 11.551, *p* < 0.001; 35–44: OR = 2.080, 95% CI = 1.302, 3.323, *p* = 0.002). Girls with household heads aged in the 15–24 and 25–34 groups had higher odds of adolescent pregnancy compared to those with household heads aged 45 and older. Higher odds of adolescent pregnancy in Nigeria were found among girls with household heads aged in the 15–24 and 25–34 groups compared to those with household heads aged 45 and older. Although girls with younger male household heads are at an increased risk nationally, those living in the rural areas with younger household heads are at an even higher risk for adolescent pregnancy. Therefore, levels of socioecological model must be considered in planning for effective interventions.

## Introduction

Adolescent pregnancy has been a major public health concern both in Nigeria and internationally for many years [1–3]. Babies of adolescent parents are at a higher risk of neonatal death [4]. Adolescent girls aged 15–19 years are two times more likely to die from pregnancy-related complications than their counterparts aged 20 years and older [4]. Globally, pregnancy-related complications are the main cause of death for women aged 15–19 years, especially in developing countries where overall maternal mortality is 400 times higher, compared to developed countries, resulting in about 700,000 deaths each year in low- and middle-income countries [5–7].

In addition, adolescent mothers and their babies are at higher risk for contracting sexually transmitted diseases (STDs), including human immunodeficiency virus (HIV) [6,7]. Data collected in 2018 show that 19% of Nigerian girls aged 15 to 19 years have reported pregnancy [8] compared to 23% in 2013 [8], a decrease of 4% in 5 years. This is a very low return on investment that can easily be surpassed by the projected widespread increase in adolescent pregnancy as a result of continued population growth in Nigeria [9]. Nigeria will need a rapid and sustained decrease for decades to be able to dispel being looked upon as one of the world’s "hot spot" for adolescent pregnancy [7,10]. Approximately 14% of the world’s maternal deaths occur in Nigeria, and 15 to19-year-old girls bear 30% of this burden of deaths [7,11].

Research shows that several sociocultural, economic, and societal factors contribute to the risk of pregnancy among girls in Nigeria, including, early-life exposure to economic hardship [9], no or low level of education [12], sexual debut between ages 15 and 19 years [13,14], being from the northern Nigeria (region), being from Ibo/Igbo (ethnicity), currently married, and currently working [14]. Additional adolescent pregnancy risk factors have included, households headed by males in the northern region, poor household in all regions, exposure to media (watching television and reading) except north-east region [15], and child marriage [16]. Further, interpersonal level factors such as traditional norms empower household heads to force child marriage due to pregnancy or household poverty [7,16]. Most especially, research shows that adolescents living in rural areas of the world are more than twice as likely to experience a pregnancy compared to those living in urban settings [7,17].

In Nigeria, despite the outlaw of induced abortion, abortions remain very high for 15 to 19-year-old girls due to unintended pregnancy [18,19]. A southwestern Nigerian study (*n* = 84) found that 39.3% of those with secondary education had unsafe abortion [19]. Child marriage contribution to adolescent pregnancy in Nigeria [16] is exacerbated by other interpersonal level factors such as cultural norms that allows household decision makers to break defilement laws with little or no consequences [7,16,20]. Hence, adolescent pregnancy remains a serious family concern because of its effect on health outcomes and family cohesion from the induced stigma [21–23].

Adolescent pregnancy in Nigeria, although well studied, has not fully recognize household head age as an important interpersonal and familial risk factor contributing to the issue of adolescent pregnancy [7]. Adolescent pregnancy is likely to be exacerbated and continue as a major public health problem based on the increase media report of rape, incest, and defilement, coupled with the projected increase in population. Cases of rape and defilement of minors became endemic in Nigeria as of 2020. Based on these reports and previously published studies, our hypothesis was that girls who lived with younger male household heads would experience the highest level of adolescent pregnancy during the research period. To the author’s knowledge, currently there are no up-to-date data as to how age of household heads contributed to the persistent problem of adolescent pregnancy in Nigeria among 15–19 years old girls between 2013 and 2018. Current research was undertaken to close this gap in relevant knowledge and make suggestions to practice going forward.

## Methods

We performed a secondary data analysis using the 2018 Nigeria Demographic and Health Survey (NDHS) data that were collected with a cross-sectional survey design. The survey was carried out between August 14, 2018 and December 29, 2018, using structured questionnaires. Data from a cohort of adolescents surveyed as part of the NDHS were used in this study to identify if household head age may be associated with adolescent pregnancy rates among survey participants.

### Socioecological model

**Fig 1:**
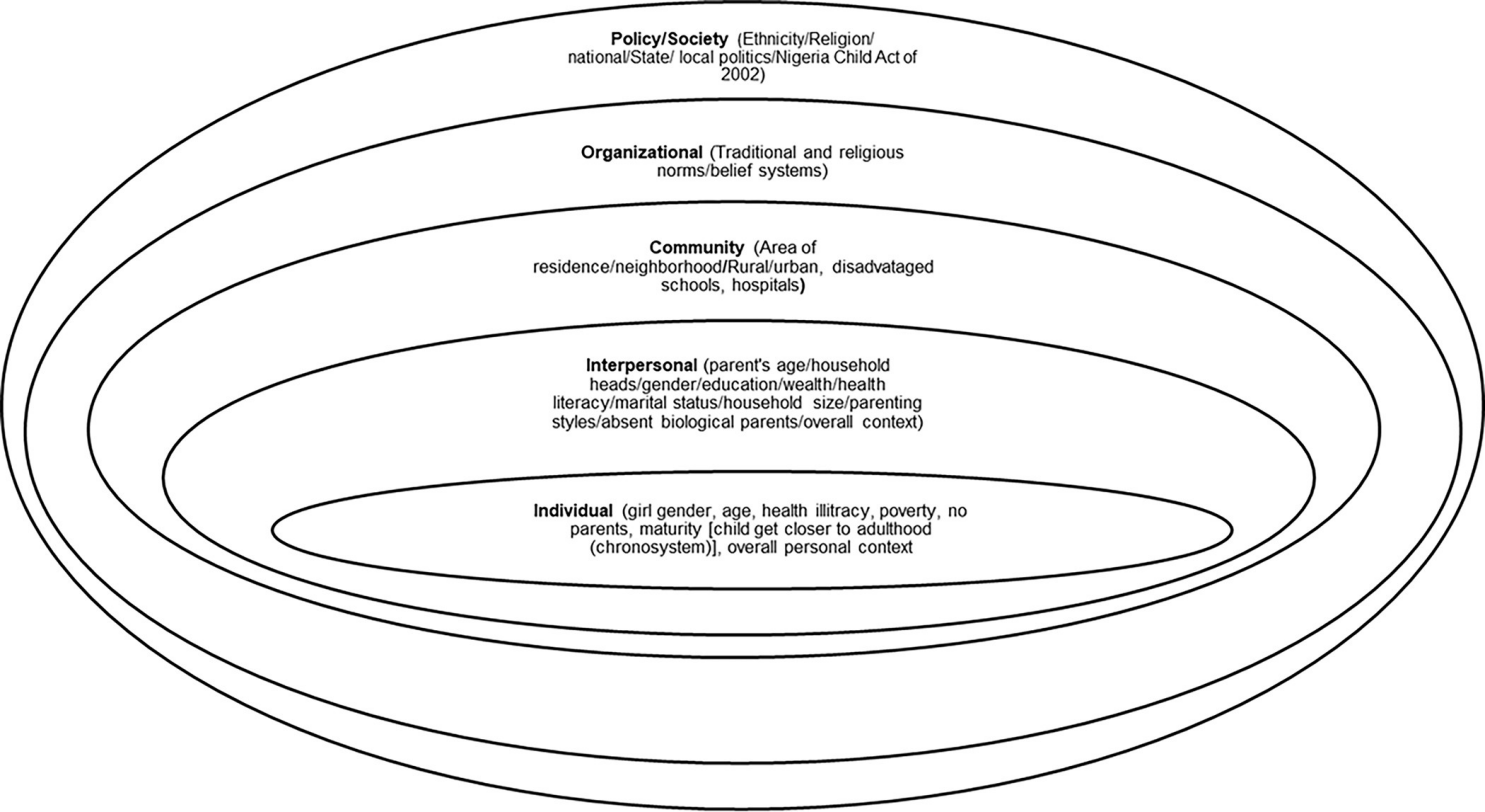
The social ecological model. Adapted from the Centers for Disease Control and Prevention (CDC). http://www.cdc.gov/violenceprevention/overview/social-ecologicalmodel.html. (Retrieved April 1, 2023).

Our study used a socioecological model (SEM) (Fig 1) to explore the relationship between family level factors and adolescent pregnancy in Nigeria, particularly how household heads age may have contributed to the level of adolescent pregnancy in Nigeria between 2013 and 2018. Understanding specific, family-based factors that influence adolescent pregnancy in Nigeria will help policymakers, practitioners, and related institutions to understand modifiable risk factors at specific socioecological levels [7].

SEM is a model that understands the entire situation [23]; hence, practitioners and policy makers can finally understand how intertwined, interconnected, or complex the risk and protective factors for adolescent pregnancy can be.

Kalinowski in 2017 [24] stated that SEM is a great model to use to explain interaction between a person and their physical, social, and political environment, including how these factors influence health outcomes. In addition, Brofenbrenner [25] and Mbulu [7] explained that SEM can be used to explore how a person and their settings (family, community, school, and society) as whole influence each other, especially during human developmental stages.

The intrapersonal level of the SEM houses personal characteristics that place adolescents at risk for pregnancy, including gender, age, health illiteracy, poverty, no parent(s), maturity, lack of knowledge, attitude, beliefs, and development stages [26]. In addition, development stage like early menarche places adolescents at higher risk for early sexual debut that can results to unwanted pregnancy, and in turn give household heads the opportunity to force child marriage [26], a frequent occurrence in Nigeria. As earlier alluded to, household heads belong in the interpersonal level of the SEM, and the current study focused on how household head age influenced adolescent pregnancy during the study period. Other factors at the interpersonal level are parents, household heads, family wealth, schools, teachers, peers, health care providers, friends, health literacy, marital status, household size, parenting style, beliefs, attitudes, and family norms. In contrast, policies enacted at the policy level of the SEM must interact or filter through three levels (organizational, community, and interpersonal) before reaching the intrapersonal level, therefore, planners must be attentive to policy alignment for any intervention to be effective, especially at the rural environment [27,28].

The major implication for the understanding of the whole situation from the SEM lens is that adolescent reproductive health practitioners, policy makers and other stakeholders must be able to identify the precise entry point for interventions and, work together to improve adolescent sexual and reproductive health [29]. We believe that a more precise or targeted type of evidence-based intervention will help reduce the level of adolescent pregnancy in Nigeria.

### Sampling

The 2018 NDHS [8] included all women aged 15–49 years in the sample households, including those who were permanent residents of the households and visitors who stayed in the household the night before. NDHS adopted the two-stage stratified cluster sampling, which allowed for the use of 2006 census pre-existing units (clusters) in place of the sampling frame. Two steps of the multistage cluster sampling technique were employed.

In this first stage, the interviewer for the NDHS randomly selected 1,400 enumeration areas (EA) with likelihood of corresponding to EA size (number of households residing in each EA). In the second stage, they selected 30 households from every cluster with equal chance of being selected, resulting to a total sampling size of about 42,000 households. However, the samples were not proportionally allocated across the states and territory of Nigeria, as this would have resulted to differences in response rates; hence, sampling weights were calculated and applied. This technique allowed results to be representative of national data. After secondary cleansing of the sample at the field level due to 11 of the 4,200 being eliminated because of a breakdown of law and order that took place while the fieldwork was in effect, the survey was successfully administered to 1,389 clusters.

### Nigeria demographic and health survey

Regarding the questionnaires [8], five varying questionnaires were distributed after being translated into the three major Nigerian languages (Hausa, Ibo, and Yoruba). The questionnaires included sociodemographic questions that collected data on variables such as age, sex, household head (name, age, and gender), type of place of residence (urban or rural), income, level of education, wealth index, ethnicity, religion, and marital status; collection of income data in NDHS surveys is a composite measure of a household’s cumulative living standard. Data collected on adolescent girls include age at the time of the survey, age at first sex, age at first child, whether currently pregnant, history of abortion, type of residence, household heads, ethnicity, religion, wealth index, and educational level. Income as wealth index was categorized as poorest, poorer, middle, richer, and richest. Respondents included in the current analysis were 15 to19-year-old Nigerian girls that at the time of the survey were either pregnant, had their first child at age 15 to 19 years, or had terminated pregnancy during these adolescent years. Excluded are all girls below age 15 years and over age 19 years.

### Statistical analysis

We used IBM SPSS statistics subscription version 28 for statistical analysis. Complex sample procedures were used in all analyses. Initial assessment of data was done using frequencies and percentages of categorical variables. The chi-square test or *t*-test for weighted cases was used to examine the relationships between independent variables and adolescent pregnancy outcomes. Variables (residence type; education, wealth index, religion, and ethnicity) which were statistically significantly associated with adolescent pregnancy at the bivariate analysis were controlled in the complex sample multiple logistic regression model to assess the effect of household head age on the adolescent pregnancy status in Nigeria during the study period. A *p*-value less than 0.05 was considered statistically significant.

### Ethics

This is a secondary data analysis that utilized the publicly available dataset from MEASURE DHS (link: The DHS Program—Nigeria: Standard DHS, 2018) which secured the ethical requirements before the survey was administered. The first author of current study, however, emailed MEASURE DHS, Rockville Maryland, USA, and was given permission to download the 2018 dataset for the purpose of this study. Therefore, no further ethics approval was needed. However, NDHS was approved by Inner City Fund (ICF) International, USA ethics committee, and collaborated with National Health Research Ethics Committee of Nigeria (NHREC), Nigerian Federal Ministry of Health ethics committee and the National Population Council of Nigeria [8]. Further, NDHS ensures that anonymity and privacy were protected by employing a coding system to avoid use of participants’ names, addresses, households, or communities [8]. NDHS staff and researchers did not have access to the code file [8].

## Results

### Population characteristics

The sociodemographic characteristics of the research variables are shown in Table 1. At the intrapersonal level (microsystem), 8,448 adolescents aged 1519 years were included in the study, of which about 25.8% (2,182) were uneducated. A little over 10% (881) were educated at the primary school level, 61.1% (5,162) were educated at the secondary school level, and 2.6% (224) had received higher education. Concerning household heads (mesosytems) 82% (6,929) were male, while 18% (1,549) were female. Approximately 7% of household heads aged 15–24 (n = 615, 7.3%) and 14.7% of those were in the 2534 age group (n = 1230). At the community level (exosystem), about 45.1% (3,813) were urban residents with 54.9% (4,635) being rural residents. As for religion, Islam accounted for more than one-half of the respondents 57.8% (4,879). There were five major wealth index categories: poorest (18.1%), poorer (20.0%), middle (20.8%), richer (20.6%), and richest (20.4%). Ethnicity varied greatly, adolescents of Hausa origin accounted for 33.6% (2,837), Igbo/Ibo was 13.9% (1,176), and other ethnic groups combined (including Yoruba) accounted for 52.5% (4,433).

**Table 1:** Demographic characteristics of the study population (weighted n = 8448).

Characteristic	Weighted *n* (%)
**Residence type**	
Urban	3,813 (45.1)
Rural	4,635 (54.9)
**Education**	
No education	2,182 (25.8)
Primary	881 (10.4)
Secondary	5,162 (61.1)
Higher	224 (2.6)
**Household head sex**	
Male	6,929 (82.0)
Female	1,519 (18.0)
**Teen pregnancy**	
Yes	1,650 (19.5)
No	6,799 (80.5)
**Wealth index**	
Poorest	1,531 (18.1)
Poorer	1,694 (20.0)
Middle	1,759 (20.8)
Richer	1,738 (20.6)
Richest	1,726 (20.4)
**Religion**	
Islam	4,879 (57.8)
Others^1^	3,569 (42,2)
**Ethnicity**	
Housa	2,837 (33.6)
Igbo	1,176 (13.9)
Others^2^	4,433 (52.5)
**Household head age**	
15–24	615 (7.3)
25–34	1230 (14.6)
35–44	1566 (18.5)
> = 45	5036 (59.6)

^1^Catholic, Non-Catholic Christians, and Traditional.

^2^Yoruba, Ekoi, Fulani, Ibibio, Igala, Ijaw, Kwanuri, and Tiv.

### Teen pregnancy by study variables

We analyzed teen pregnancy by study variable (Table 2) to examine whether the participants’ family level variables, particularly household head age, were associated with adolescent pregnancy in Nigeria. A cross tabulation table with chi-square tests was conducted on household head age and adolescent pregnancy. The household head age variable was transformed from interval/ratio to an ordinal variable. Household head age was found to have a significant association with adolescent pregnancy aged 15–19 years (*p* < 0.001). Adolescent pregnancy rates were significantly higher in the younger household heads age groups (p < .001). Adolescent pregnancy rates were 58.5% in households with heads aged 15–24 and 62.9% in households with heads aged 25–34.

**Table 2:** Percent adolescent pregnancy per variable type (weighted n = 8448).

Variable type	Yes *n* (%)	No *n* (%)
**Residence type** ***		
Urban	335 (8.8)	3,478 (91.2)
Rural	1,314 (28.4)	3,320 (71.6)
**Education** ***		
No education	996 (45.6)	1,186 (54.4)
Primary	214 (24.3)	667 (75 > 7)
Secondary	436 (8.4)	4,725 (91.6)
Higher	4 (1.8)	220 (98.2)
**Household head sex** ***		
Male	1,488 (21.5)	5441 (78.5)
Female	161 (10.6)	1,358 (89.4)
**Wealth index** ***		
Poorest	404 (26.4)	1,127 (73.6)
Poorer	392 (23.2)	1,301 (76.8)
Middle	416 (23.6)	1,343 (76.4)
Richer	303 (17.4)	1,436 (82.6)
Richest	134 (7.8)	1,598 (92.2)
**Religion** ***		
Islam	1,279 (26.2)	3,600 (73.8)
Others^1^	371 (10.4)	3,199 (89.6)
**Ethnicity** ***		
Housa	855 (30.1)	1,982 (69.9)
Igbo	99 (8.4)	1,077 (91.6)
Others^2^	696 (15.7)	3,737 (84.3)
**Household head age** ***		
15–24	359 (58.5)	255 (41.5)
25–34	773 (62.9)	456 (37.1)
35–44	234 (14.9)	1332 (85.1)
> = 45	282 (5.6)	4754 (94.4)

***Indicates *p* < 0.001 from chi-square test.

^**1**^Catholic, Non-Catholic Christians, and Traditional.

^2^Yoruba, Ekoi, Fulani, Ibibio, Igala, Ijaw, Kwanuri, and Tiv.

### Multiple logistics regression results

Results of the multiple logistic regression (Table 3) demonstrate significant interaction effects between household head age and sex (*p* < 0.001) after adjusting for residency type, education level, household head sex, wealth index, religion, and ethnicity. Girls with male household heads aged 45 and older had lower odds of adolescent pregnancy (OR = 0.619; 95% CI = 0.447, 0.856; *p* = 0.004) compared to those with female household heads in the same age group. However, girls with male household heads in three younger age groups had higher odds of adolescent pregnancy than those with female household heads in the corresponding age group (15–24: OR = 1.719, 95% confidential interval (CI) = 1.042, 2.835, p = 0.034; 25–34: OR = 4.790, 95% CI = 1.986, 11.551, *p* < 0.001; 35–44: OR = 2.080, 95% CI = 1.302, 3.323, *p* = 0.002). Girls with household heads aged in the 15–24 and 25–34 groups had higher odds of adolescent pregnancy compared to those with household heads aged 45 and older.

**Table 3:** Final multiple logistics regression model for study variables and pregnancy outcome in Nigerian adolescents aged 15–19 years between 2013 and 2018.

	B				95% CI	
		Std Error	*p*-value	OR	Lower	Upper
**Residency Type** ^**a**^						
Urban	-0.503	0.117	< 0.001	0.605	0.481	0.761
**Education** ^**b**^			<0.001			
No education	3.003	0.562	< 0.001	20.150	6.691	60.683
Primary	2.525	0.560	< 0.001	12.490	4.162	37.485
Secondary	1.811	0.544	< 0.001	6.117	2.104	17.788
**Religion** ^**c**^						
Others	0.042	0.129	0.743	1.043	0.810	1.343
**Ethnicity** ^**d**^			0.389			
Hausa	0.123	0.109	0.259	1.130	0.914	1.399
Igbo	0.129	0.162	0.425	1.138	0.828	1.565
**Household head sex** ^**e**^						
Male	-0.480	0.166	0.004	0.619	0.447	0.856
**Wealth index** ^**f**^			0.012			
Poorest	0.395	0.180	0.028	1.485	1.043	2.115
Poor	0.304	0.171	0.074	1.356	0.970	1.894
Middle	0.556	0.165	<0.001	1.743	1.260	2.411
Richer	0.499	0.172	0.004	1.647	1.176	2.306
**Household head age** ^**g**^			<0.001			
15–24	1.992	0.269	<0.001	7.332	4.323	12.433
25–34	0.976	0.461	0.034	2.655	1.075	6.559
35–44	-0.029	0.267	0.914	0.972	0.575	1.641
**Household head age by sex**			<0.001			
Male*15–24	1.021	0.303	<0.001	2.777	1.533	5.030
Male*25–34	2.046	0.472	<0.001	7.739	3.068	19.521
Male*35–44	1.212	0.287	<0.001	3.361	1.914	5.902

Reference category: a- rural; b- higher education; c- Islam; d- others; e- female; f- richest; g- 45+.

## Discussion

Our study used a SEM to explore how household head age contributed to the persistent adolescent pregnancy in Nigeria, particularly during the study period between 2013 and 2018. This study demonstrates that the independent variables of interest (household head age at the SEM interpersonal level) examined for this study was associated with higher risk of adolescent pregnancy (intrapersonal level characteristics) in Nigeria aged 15–19 years during the study period; illustrating that this factor should be targeted with SEM-based preventive programs that allow intervention planners to take into account how community, organizational, and policy levels could also contribute to or diminish success of interventions [27]. Furthermore, the entry point for the intervention relative to household head age is evident at age group with higher prevalence of adolescent pregnancy: younger household heads (15–24 (58.5%) and 25–34 (62.9%). Another study [22] had similar results but with lower prevalence of adolescent pregnancy: 36% and 20% among household heads age less than 30 years and 30–40 respectively. The plausible reasons for this finding could be due to country-specific differences. For example, while high level of child marriage in Nigeria might have contributed due to non-implementation of defilement laws (policy level), Rwanda as a country updated their own defilement laws to punish whoever impregnate any girl [22].

The current study also found that adolescent girls with older male household heads had lower risk of pregnancy (OR = 15–24: OR = 1.719, 95% CI = 1.042, 2.835, p = 0.034; 25–34: OR = 4.790, 95% CI = 1.986, 11.551, p < 0.001; 35–44: OR = 2.080, 95% CI = 1.302, 3.323, *p* = 0.002). This finding also agrees with Uwizeye, et al. [22] who found the lowest risk of adolescent pregnancy among girls with older household heads (45–54: OR = 0.07, 95% CI = 0.06–0.19). The possible explanation for this similarity among this age group might be due to high level of parental support (provision of financial needs and safety) for their girls despite country differences.

Further, Izugbara study [29] conducted in Nigeria found that girls who lived with younger household heads (30–44) had higher risk of pregnancy (AOR = 0.53, 95% CI = 0.4209848–0.6776125) compared to those with older (45–59: AOR = 0.21, 95% CI = 0.1542932–0.3012827) household heads. Of note, Izugbara’s study focused only on unintended pregnancy among unmarried adolescents aged 15–19 years [29]. Results of current and Izugbara studies are critical for assessing a clear entry point for any intervention. The unmarried participants (girls in the intrapersonal space of SEM) might have been poorly protected and provided for by their younger household heads in the interpersonal space [22,27]. This finding is key as to why current study was undertaken.

In addition, a 2015 South Africa study [28] investigated contraceptive use among child-headed households and found that adolescents who live in child-headed households are at higher risk of becoming pregnant. Moreover, the HIV/AIDS epidemic of the 1980s through the 1990s exposed some aspects of child-headed households; defined as a household consisting of people younger than age 18 where the oldest among them assumed the head to ensure that everyone under him or her has food and shelter, whereas, in most cases, these children are school dropouts before or after unwanted pregnancy [22,28]. These findings suggest the need to maintain at least one older household head, but when this is not possible then tailored sex education and provision of contraceptives should be made available as soon as possible [28].

### Practical and policy implications

Results from this study have health-related quality of life, cultural, practical, and policy implications for Nigeria, particularly in the highest-risk rural areas. Our findings on household head age inform all Nigerian stakeholders of the need to allocate more resources to target younger household heads across the country for home and school-based comprehensive sex education strategy [30]. Such sex education should aim to increase individual, family, and community awareness of the negative health effects of adolescent pregnancy. However, no public health intervention has worked in Nigeria for decades due to weak enforcement of policies and strategies, therefore, policy makers and practitioners should use findings such as ours on the household head age to employ policies, programs, or strategies similar to Ethiopian government’s Health Extension Workers Program [30–32]. The program employed aspects of SEM as narrated by Mbulu [7] and McLeroy, et al [33]. Similarly, funding agencies may be more open to fund well-targeted programs with focus on high-risk adolescents identified by this research. For example, the World Bank group [34] is poised to support programs that expand healthcare, and improve well-being from the bottom up (poor, underserved, and vulnerable).

### Strengths and limitations

This study has several strengths. We used a nationally representative dataset from the 2018 NDHS. Hence, our results can be generalized to adolescent girls aged 15–19 years in Nigeria in both rural and urban settings. However, the use of a secondary dataset from a cross-sectional survey exposed the study to some specific limitations [7,22]. We may not have controlled all covariates since those needed for this study may not have been collected, also, some important variables may have been deleted to conceal identity of participants; hence, not available for analysis [35,36]. In addition, we recoded categorical and continuous variables for this research, such action according to studies [37] has the potential to generate an insufficient or biased estimate. Further, recall bias among participants, particularly rural and older responders in remembering dates of current and past pregnancy might have resulted in exaggerated or underestimation of data, including misclassification of variables and distortion of association [37]. These uncertainties or biases may have resulted in faulty estimates of the variables’ effect.

### Recommendations and further research

The finding that girls with younger household heads are at higher risk for adolescent pregnancy experience indicates a troubling trend, particularly as public health officials predict higher population growth of Nigerian adolescent girls aged 10–17 years by 2030 [7,9]. Hence, we recommend a peer-led and school-based comprehensive sex education intervention [30,38]. We further recommend a home-based version of the sex education program to focus on young male household heads and increase awareness of the need to comply with legislative actions meant to deter any marriage before the age of 18 [32]. However, many states in Nigeria are not enforcing such laws [38].

Most literature on adolescent pregnancy in Nigeria has used data from NDHS [16,39]; future studies should focus on Nigerian villages that may not have been reached by sexual reproductive health research or services; insiders’ perspective and key informant interview should be employed for in-depth knowledge on lived experiences of pregnant and nonpregnant adolescent girls, and that of key informants [7,32]. Information from such a study could provide data for the development of better and effective health promotion interventions for adolescent pregnancy precisely for a particular village [14,39].

## Conclusion

The persistent level of adolescent pregnancy in Nigeria is associated with multifaceted factors. With the use of socioecological framework, this study, however, identified living with a younger age male household head behavior (a component of interpersonal level) as one of the modifiable risk factors to address. Understanding both the intrapersonal and interpersonal variables that contribute to the persistent of adolescent pregnancy in Nigeria requires comprehensive collaborative efforts involving all five levels of the SEM. Further, evaluation of the community, policy, and care delivery that interact with the intrapersonal and interpersonal variables is essential for targeting intervention that are both feasible for, and acceptable by the population of interest. Therefore, equitable resource reallocation to target specific household heads in the rural areas is critical for the provision of comprehensive and unrestricted sex education to all school-aged girls, and their parents across Nigeria. Nigeria could become a model for adolescent reproductive health with an additional 5% decrease in adolescent pregnancy, and such progress could remove the “hot spot” label and other negative associations of Nigeria with high rates of adolescent pregnancies, including deaths.
